# Interface chemistry of an amide electrolyte for highly reversible lithium metal batteries

**DOI:** 10.1038/s41467-020-17976-x

**Published:** 2020-08-21

**Authors:** Qidi Wang, Zhenpeng Yao, Chenglong Zhao, Tomas Verhallen, Daniel P. Tabor, Ming Liu, Frans Ooms, Feiyu Kang, Alán Aspuru-Guzik, Yong-Sheng Hu, Marnix Wagemaker, Baohua Li

**Affiliations:** 1 Shenzhen Key Laboratory of Power Battery Safety and Shenzhen Geim Graphene Center, Tsinghua Shenzhen International Graduate School, Tsinghua University, Shenzhen, 518055 China; 2 School of Materials Science and Engineering, Tsinghua University, 100084 Beijing, China; 3 Department of Chemistry and Chemical Biology, Harvard University, Cambridge, MA 02138 USA; 4 Key Laboratory for Renewable Energy, Beijing Key Laboratory for New Energy Materials and Devices, Beijing National Laboratory for Condensed Matter Physics, Institute of Physics, Chinese Academy of Sciences, 100190 Beijing, China; 5 Department of Radiation Science and Technology, Delft University of Technology, Mekelweg 15, 2629 JB Delft, The Netherlands; 6 Department of Chemistry and Department of Computer Science, University of Toronto, Toronto, ON M5S 3H6 Canada

**Keywords:** Chemistry, Engineering

## Abstract

Metallic lithium is a promising anode to increase the energy density of rechargeable lithium batteries. Despite extensive efforts, detrimental reactivity of lithium metal with electrolytes and uncontrolled dendrite growth remain challenging interconnected issues hindering highly reversible Li-metal batteries. Herein, we report a rationally designed amide-based electrolyte based on the desired interface products. This amide electrolyte achieves a high average Coulombic efficiency during cycling, resulting in an outstanding capacity retention with a 3.5 mAh cm^−2^ high-mass-loaded LiNi_0.8_Co_0.1_Mn_0.1_O_2_ cathode. The interface reactions with the amide electrolyte lead to the predicted solid electrolyte interface species, having favorable properties such as high ionic conductivity and high stability. *Operando* monitoring the lithium spatial distribution reveals that the highly reversible behavior is related to denser deposition as well as top-down stripping, which decreases the formation of porous deposits and inactive lithium, providing new insights for the development of interface chemistries for metal batteries.

## Abstract (web summary)

Interface chemistry is essential for highly reversible lithium-metal batteries. Here the authors investigate amide-based electrolyte that lead to desirable interface species, resulting in dense Li-metal plating and top-down Li-metal stripping, responsible for the highly reversible cycling.

## Introduction

Lithium-ion batteries (LIBs) have enabled the progress in portable electronics and automotive applications^1,2^. However, the demand for higher energy density batteries, requires new electrochemical energy storage technologies beyond the current Li-ion insertion chemistries^3^. For decades metallic Li has been considered as promising anode owing to its high specific capacity of 3860 mAh g^−1^ and low redox potential of −3.04 V (vs. the standard hydrogen electrode), providing a more than ten-time larger specific capacity in comparison with the current graphite anode^4^. However, several challenges remain to be addressed before Li-metal batteries can be applied in practice, including the uncontrolled dendrite growth and low Coulombic efficiency, which are responsible for the poor cycling stability and safety hazards^5–7^. The key issue is the high reactivity between metallic Li and organic liquid electrolytes, producing decomposition species that cover the surface of the electrodes, which is referred to as solid electrolyte interphase (SEI)^2,8,9^. The SEI layer can support the reversible cycling of electrodes by passivating detrimental reactions, however, extensive and uncontrolled SEI growth leads to irreversible electrolyte and electrode degradation reactions. The cycling reversibility, and hence of the battery cycle life depends on the properties of the SEI species. Electrolyte reduction products are often poor ion-conducting inorganic constituents (e.g., Li_2_O, LiF, and Li_2_CO_3_) and unstable organic constituents (e.g., ROCO_2_Li and ROLi, R representing −H, CH_3_, −CH_2_CH_3_, etc.). These species can result in the continuous consumption of both electrolyte and Li metal, and hinder the reversible Li plating/stripping by increasing the interface impedance^5,6,10,11^.

A good SEI layer will serve as a physical barrier that blocks further side reactions between metallic Li and organic liquid electrolytes, where its composition should facilitate dense, uniform and reversible plating/stripping^2,8,9^. The composition of the SEI layer is determined by the electrolyte chemistry, including the selection of salts, solvents, additives and so on^12–16^. To realize the ideal SEI components, having negligible electronic conductivity, a high ionic conductivity and good mechanical/chemical stability, various strategies have been developed^12,17–21^. Highly or locally concentrated electrolytes are used to enhance the electrochemical stability towards Li metal anodes^12,17,18,22^. Increasing the salt concentration will effectively increase the interactions between cations and anions in the liquid electrolyte environment, and minimize the presence of free-electron-state solvent molecules, thereby reducing decomposition of the solvents. However, the use of high salt concentrations introduces other challenges, such as poor ionic conductivity, high viscosity and high cost, which need to be addressed towards practical applications^19,23^. Highly fluorinated electrolytes, present another promising strategy based on its high stability against both reduction and oxidation in high-voltage systems^17,19^. All-fluorinated electrolytes with high salt concentrations (up to 7 M)^17^ and low salt concentration (1 M)^19^ have been investigated, demonstrating improved cycling stability for the LiNi_0.5_Mn_1.5_O_4_ (5 V) and LiCoPO_4_ (4.8 V) high-voltage cathodes, respectively. Despite these promising results, the concern is the formation of poor ionic conducting fluoride species, especially inorganic LiF^24^, that increase the interface impedance at both the anode and the cathode. In addition, many efforts have been devoted to ether-based electrolytes due to their lower reactivity with Li metal, as compared to carbonate electrolytes. The ester groups typically promote a multi-state decomposition, leading to further reaction and dissolution of the existing SEI components^10,15,20,25–27^. Unfortunately, the limited oxidation stability of ether-based electrolytes (<4 V vs. Li^+^/Li) precludes their application in combination with high-voltage layered LiCoO_2_ or Li(NiCoMn)O_2_ cathodes^19,28^. Recently, an ether electrolyte with a concentrated 2 M lithium bis(trifluoromethanesulfonyl)imide (LiTFSI) and 2 M lithium difluoro(oxalato)borate (LiDFOB) salts has been reported, demonstrating good interface stability towards a high-voltage LiNi_1/3_Mn_1/3_Co_1/3_O_2_ cathode and the Li metal anode^18^. These advances are very promising, yet challenges remain, motivating further electrolyte innovations that promote highly reversible Li metal batteries and fundamental studies towards the understanding of Li^+^ plating/stripping behavior and its relation to the interface chemistry.

Herein, we report an electrolyte that demonstrates excelent cycling stability towards both the Li-metal anode and the Ni-rich NCM811 cathode, based on the rational design of the interface chemistry towards highly ion-conductive and stable interface species. Amide compounds, such as *N, N*-dimethylformamide (DMF) and dimethylacetamide (DMA), are similar to carbonate solvents, where the difference is the substitution of the –OR_2_ ester group with the –NR_2_ amide group. Compared to the widely used ethylene carbonate (EC) and dimethyl carbonate (DMC) solvents, amide solvents are expected to form less low ion-conductive inorganic constituents, such as Li_2_O and Li_2_CO_3_, due to absence of the ester groups. Amide compounds have been used as additive to increase the chemical stability against reduced superoxide in Li-air batteries^29^, however, systematic exploration of amides as solvents for Li metal batteries not been investigated to date. In this work, a 1 M Lithium bis(trifluoromethanesulfonyl)imide (LiTFSI) in a mixture of 2,2,2-Trifluoro-*N, N*-dimethylacetamide and fluoroethylene carbonate (FDMA: FEC, 1:1 by volume) was prepared to explore its performance in Li-metal batteries. A high Coulombic efficiency of ~99.3% is achieved for Li plating and stripping in Li||Cu cells, and stable Li plating/stripping is also observed during the prolonged cycling of symmetric Li||Li cells. In contrast to the conventional electrolyte system, 1 M LiPF_6_-EC/DMC, the Li stripping occurs at the top of the Li-metal deposit, providing new insights in the prerequisites of highly reversible lithium metal batteries. Excellent cycling stability is demonstrated in full cells with Ni-rich LiNi_0.8_Co_0.1_Mn_0.1_O_2_ (NCM811) as cathode, having a high-mass loading of 3.5 mAh cm^−2^. Quantum chemistry calculations and experimental measurements provide detailed insights in the interface chemistry that is responsible for the improved performance of this promising electrolyte.

## Results

### Screening of salts and solvents

**Fig. 1:**
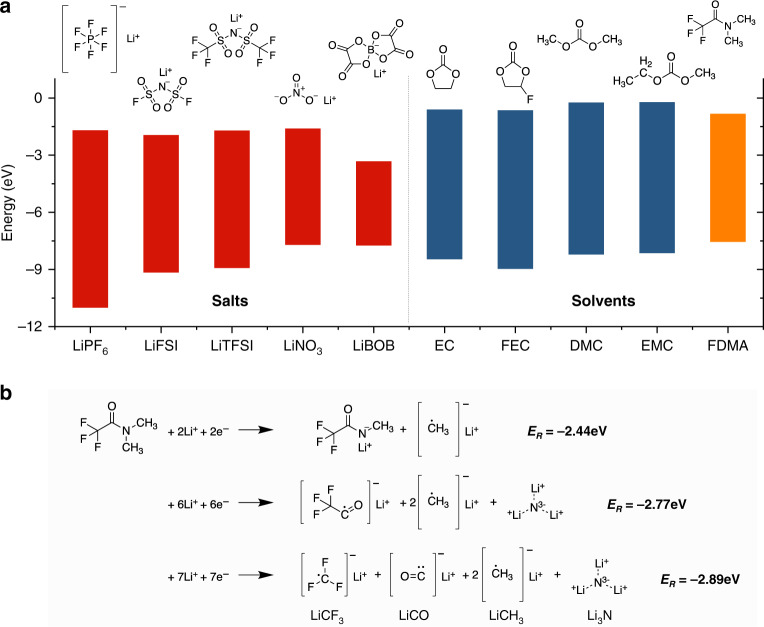
Molecular orbital energies and possible decomposition pathways. **a** Comparison of the highest occupied molecular orbital (HOMO)-lowest unoccupied molecular orbital (LUMO) energy levels for commonly used Li salts and solvents including the 2,2,2-Trifluoro-*N, N*-dimethylacetamide (FDMA). **b** Possible chemical reactions of FDMA on Li-metal surface according to the reaction energy.

At the onset of SEI formation, the solvent sheet with Li^+^ will be reduced by the electrons accumulated in the vicinity of Li metal surface, resulting in the SEI components. To control the SEI composition requires that the preferred interface species should have a higher reduction potential as compared to the main solvents, which is equivalent to a higher electron affinity or a lower lowest unoccupied molecular orbital (LUMO)^30^. According to the LUMO energy obtained from density functional theory (DFT) simulations, shown in Fig. 1a and Supplementary Table 1, FDMA exhibits the lowest LUMO among all solvents studied, demonstrating a high electron affinity, which can be expected to decompose first and hence dominate the SEI formation. LiNO_3_, as a N-donating additive, has been widely used in ether-based electrolytes^31–33^, however, it is practically insoluble in carbonate solvents (<10^−5^ g mL^−1^) making this a challenging strategy for practical application^21,34,35^. Although FDMA has a comparable HOMO-LUMO gap to LiNO_3_, FDMA has the advantage of being highly soluble in both ether and carbonate solvents, providing a more practical route to introduce N-containing components in the SEI. Since FDMA has a relatively high HOMO level, the utilization of FEC as co-solvent with the lowest HOMO level among the widely used solvents is necessary to enhance the high-voltage stability towards the cathode by means of the formation of LiF^17,19^. Considering the Li salts, LiPF_6_ has the advantage of having a large HOMO-LUMO gap, however, FEC is found to be thermally instable in LiPF_6_-based electrolytes, which will trigger the generation of unwanted HF and various acids. These acids will cause significant dissolution of transition metal ions into the electrolytes, and lead to the serious degradation of cathode materials^36,37^. Other frequently used salts, having a similar electrochemical window, are LiTFSI and LiFSI, of which LiTFSI is reported to be more stable towards Li metal due to the stable –CF_3_ group^38^. According to the discussion above, we prepared 1 M LiTFSI in a solvent mixture of FDMA/FEC (1:1 by volume) as an electrolyte and explored its properties in Li-metal batteries.

To study the role of the FDMA in the SEI formation, possible decomposition mechanisms of FDMA were considered based on reaction energy calculations. Three reactions, including no Li^+^/electron attack, single Li^+^/electron reactions, and two Li^+^/electron reactions, are considered in combination with the five possible bond-breaking reactions as shown in Supplementary Fig. 1. The thermodynamically most likely first step of the FDMA decomposition is that two Li^+^ and two electrons attack the N–CH_3_ bond to form CH_3_Li and a N-containing species. In this amide molecule, the lone pair electron of the amino nitrogen will be conjugated with the π electron from the carbonyl group, in which the electron cloud on the nitrogen is less dense and therefore prone to accept an electron. As a result, we propose a possible three-step decomposition mechanism as shown in Fig. 1b and Supplementary Fig. 2. It is worth noticing that the decomposition of FDMA will produce Li_3_N in the second step, and that the final decomposition products are small molecule compounds which are unlikely to undergo further reaction. Furthermore, we calculated the HOMO-LUMO window of DMA and DMF for comparison as shown in Supplementary Fig. 3. The result shows that the introduction of –CF_3_ group reduces both the LUMO and HOMO levels, enabling FDMA to participate in the formation of SEI layers at higher potentials. Meanwhile, the stronger electron-withdrawing –CF_3_ group, as compared to that of –H and –CH_3_ groups, generates a repulsive force towards the C=O dipole, which contributes to an improved oxidation stability^39^.

### Electrochemical stability

**Fig. 2:**
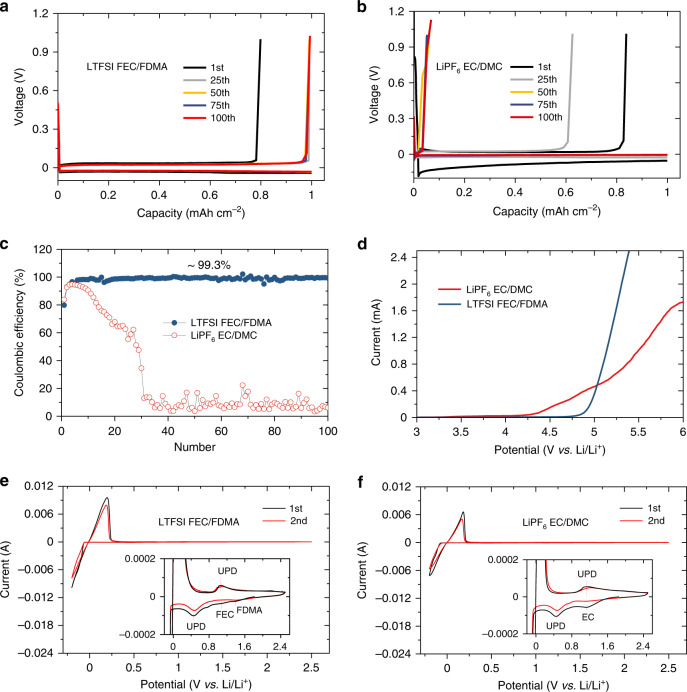
Electrochemical properties of the electrolytes. **a**, **b** Voltage profiles for Li plating/stripping on a Cu working electrode cycled in 1 M LiTFSI-FEC/FDMA and 1 M LiPF_6_-EC/DMC at a current density of 1 mA cm^−2^. **c** Li plating/stripping Coulombic efficiency in Li||Cu cells using different electrolytes. Li was electrodeposited at 1 mA cm^−2^ to a total capacity of 1 mAh cm^−2^ followed by stripping to a cut-off voltage of 1.0 V vs. Li^+^/Li. **d** Positive linear sweep voltammograms (LSV) sweep to gauge the oxidation stabilities of the different electrolytes as evaluated on Li||stainless-steel cells at a scanning rate of 5 mV s^−1^. **e**, **f** Cyclic voltammetry (CV) curves of Li||Cu cells in 1 M LiTFSI-FEC/FDMA and 1 M LiPF_6_-EC/DMC electrolytes at a scanning rate of 1 mV s^−1^. The insets show the enlarged view regions.

Li||Cu cells with different electrolytes were assembled to investigate the Li plating/stripping upon electrochemical cycling as shown in Fig. 2a–c. Clearly, the as-prepared amide electrolyte, 1 M LiTFSI-FDMA/FEC (1:1 in volume), exhibits a significantly enhanced Coulombic efficiency and cycling stability as compared to that of the conventional 1 M LiPF_6_-EC/DMC (1:1 in volume) electrolyte and the reported performance of 1 M LiPF_6_-FEC/DMC (1:1 in volume)^19^. The average Coulombic efficiency of the Li plating/stripping reaches ~99.3% for the as-prepared electrolyte up to 100 cycles, which is a major improvement compared to conventional EC-based electrolytes as shown in Fig. 2c. The high reversibility of the Li plating/stripping sets in after several initial cycles, which is competitive to that observed in a concentrated fluorinated electrolyte (97.7% for the first 100 cycles)^17^. The synergy of the components in improving the cycling stability of the as-prepared amide electrolyte is confirmed by comparing the Coulombic efficiency of Li||Cu cells with different solvents and Li salts, as shown in Supplementary Fig. 4. In addition, the present amide electrolyte generates smaller overpotentials of ~65 mV against the highly fluorinated electrolyte (e.g., ~110 mV in 7 M LiFSI-FEC)^17,19^. The difference in cycling stability and overpotential is also observed in the symmetric Li-metal cells, especially at higher current densities, see Supplementary Figs. 5–8, where the amide electrolyte exhibits an overpotential of only ~10 mV and ~20 mV at a current density of 1 and 3 mA cm^−2^ for more than 1000 and 900 h, respectively, outperforming the EC-based electrolyte. Furthermore, the cycling stability at a high current density of 5.0 mA cm^−2^, and the rate test from 0.5 to 8 mA cm^−2^ further demonstrate the competitive reaction kinetics of this amide electrolyte (Supplementary Figs. 7 and 8). The high reversibility and remarkable cycling stability of the Li plating/stripping can be attributed to the interface chemistry that leads to a SEI layer in this electrolyte having the desired properties. A noticeable difference is found for the electrolytes after 100 cycles as shown in Supplementary Fig. 9, where the surface of Li metal anode in the EC-based electrolyte turns brown while in the present electrolyte retains its original color. Furthermore, electrochemical impedance spectroscopy is carried out on the Li||Li symmetric cells as shown in Supplementary Fig. 10, where the amide-based electrolyte exhibits a lower electrochemical impedance compared to the EC-based electrolyte, consistent with the above analysis.

The oxidation stability of various electrolytes was evaluated using linear sweep voltammetry (LSV) on Li||stainless-steel cells. The EC-based electrolyte shows a lower oxidation potential, as evidenced by a rapid increase in current above ~4.1 V as shown in Fig. 2d, while for the present amide-based electrolyte, the oxidation sets in at much higher potential. Cyclic voltammetry (CV) test of Li||Cu cells were performed to study the reductive stability as shown in Fig. 2e, f and Supplementary Fig. 11. It is observed that FDMA has a slightly higher reduction potential than that of EC and FEC. After several initial cycles, the reduction peaks disappear as shown in Supplementary Fig. 12, indicating that FDMA and FEC are only decomposed during the initial cycles.

### Evolution of morphology for Li-metal anode

**Fig. 3:**
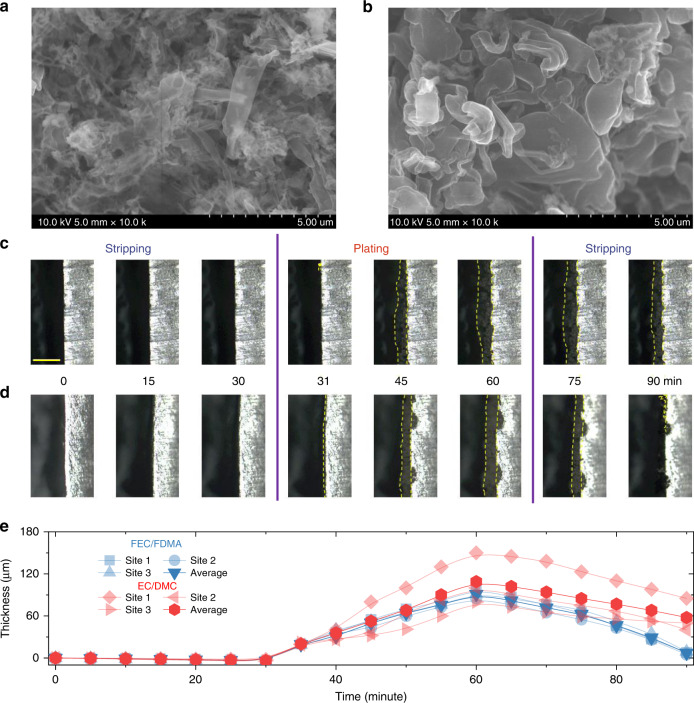
Characterization of morphology during Li^+^ plating/stripping. **a**, **b** Scanning electron microscopy (SEM) images after Li plating at current density of 1 mA cm^−2^ to the capacity of 1 mAh cm^−2^ on a Cu substrate in 1 M LiPF_6_-EC/DMC and 1 M LiTFSI-FEC/FDMA. **c**, **d** In situ optical microscopy images showing the cross-section of Li electrode upon plating/stripping in a symmetric cell at 1 mA cm^−2^, recorded at the specified plating/stripping stage in a 1 M LiPF_6_-EC/DMC and 1 M LiTFSI-FEC/FDMA electrolytes. The scale bar for **c**, **d** is 200 μm. **e** The evolution of thickness for the deposition of during Li plating/stripping. Three locations, at 1/4, 1/2, and 3/4 along the Li-metal foil are chosen to measure the change in thickness, from which the average thickness is calculated.

To investigate the Li deposition morphology in different electrolytes, ex situ scanning electron microscopy (SEM) and in situ optical microscopy are employed, the results of which as shown in Fig. 3a–d. After 100 cycles, the porous structure with needle-like dendritic Li for a few hundred nanometers is observed on the surface of Li metal anode after cycling in the EC-based electrolyte, as shown in Fig. 3a. In contrast, the as-prepared amide-based electrolyte leads to a compact aggregate of granular Li metal particles with sizes in the range of a few microns, as shown in Fig. 3b. The denser Li deposition will lead to less exposure of fresh electrolyte to the plated Li metal, reducing the detrimental decomposition reactions, resulting in a higher cycling stability. Furthermore, a dedicated cell is designed to monitor the Li plating/stripping in real-time, in situ, with an optical microscope. The images in Fig. 3c, d are taken at specific stages of cycling for the two different electrolytes and the corresponding movies are provided as Supplementary Movies 1 and 2, respectively. At first, the thickness evolution of the pristine Li metal was monitored during stripping at 1 mAh cm^−2^, where a slight decrease is observed for both electrolytes. Upon subsequent Li plating, protrusions appear on the surface of the Li metal electrodes in both electrolyte systems. However, in the EC-based electrolyte, uneven Li deposition is observed resulting in a porous morphology as observed from the side view. In contrast, in the amide electrolyte, a more homogeneous nucleation leads to denser Li deposition as shown in Fig. 3d. Upon subsequent stripping, almost all the Li deposits disappear in amide electrolyte, whereas a large amount of residual Li remains on the surface in the presence of the EC-based electrolyte. The corresponding evolution of Li-metal thickness during plating and stripping in both electrolytes, shown in Fig. 3e, confirms the denser and more uniform Li deposition in the amide electrolyte.

### Interface between anode and electrolyte

**Fig. 4:**
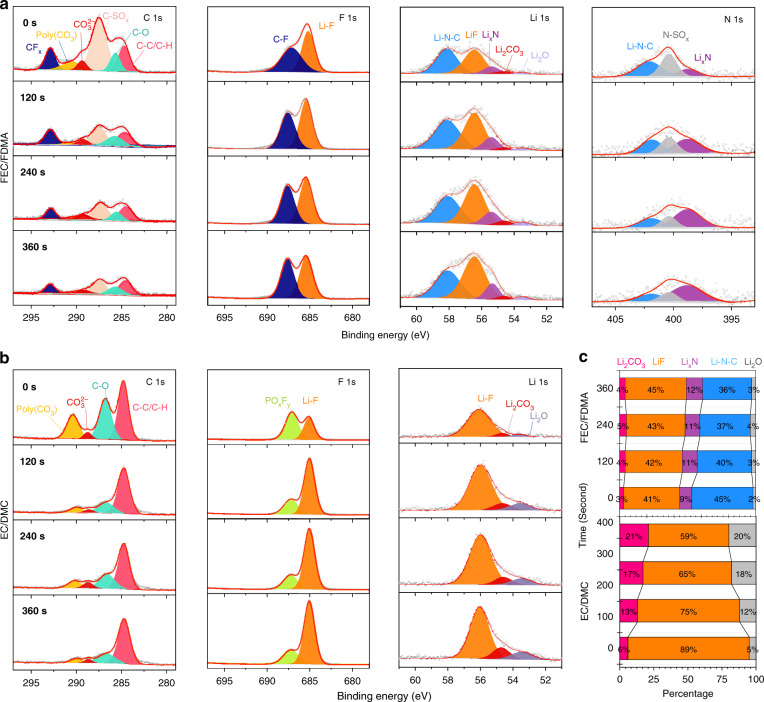
Components on the surface of anodes after cycling. **a**, **b** X-ray photoelectron spectroscopy (XPS) depth profiles of C 1s, F 1s, Li 1s and N 1s spectra of Li metal anodes after 50 cycles for both electrolytes. **c** The relative composition of Li-containing species.

To determine the interface decomposition species, X-ray photoelectron spectroscopy (XPS) analysis is performed on the cycled Li metal anodes for both the amide-based electrolyte (Fig. 4a) and the conventional EC-based electrolyte (Fig. 4b), where the survey spectra is shown in Supplementary Fig. 13. In the C 1s spectra, both electrolytes present the commonly observed species, including C–C/C–H (~284.8 eV), C–O (~285.7 eV), CO_3_^2−^ (~289.3 eV) as well as poly(CO_3_) (~290.3 eV)^40,41^. In contrast, for the amide electrolyte, two additional peaks appear that can be attributed to C–SO_x_ (~287.6 eV)^18^ and CF_x_ (~292.9 eV)^42,43^, most likely resulting from LiTFSI or FDMA. The existence of CF_x_ (~687.6 eV) is further supported by the F 1s spectra, and the other peak at ~685 eV corresponds to LiF, which suggests the preferred reaction of FEC with Li metal^44,45^. For the EC-based electrolyte, the two major peaks at ~687 and 685 eV can be attributed to fluorophosphates (PO_x_F_y_) and LiF, most likely generated via the reactions of PF_5_ and/or PF_6_ groups with Li metal, which testify the participation of LiPF_6_ in the SEI formation. For the Li 1s spectrum of the EC-based electrolyte, the peak in the range of ~53–56 eV can be deconvoluted into three components centered at ~55.7 eV, ~54.3 eV and ~53.2 eV, which can be assigned to LiF, Li_2_CO_3_ and Li_2_O, respectively^46^. For the amide system, two more peaks appear in the Li 1s spectrum, centered at ~58.1 eV and ~55.7 eV, which can be assigned to two N-containing species, Li–N–C and Li_x_N, originating from the decomposition of FDMA and LiTFSI. From the results, the relative amount of Li-containing species at different depths is determined as shown in Fig. 4c. For both systems LiF accounts for a large portion of the SEI, where for the amide system, Li–N species (including Li–N–C and Li_x_N) dominate the SEI chemistry. These Li–N species can be identified more clearly in the N 1s spectrum, where the three deconvoluted contributions (~402.1, ~400.4 and ~398.7 eV) can be assigned to Li–N–C, N–SO_x_ and Li_x_N, respectively^42,47,48^. After sputtering, the Li_x_N signal becomes more apparent, along with a decrease of the Li–N–C signal, which can be explained by the different reduction products of FDMA, depending on the amount of reacting Li^+^ and e^−^ as studied by the DFT calculations. In the inner part of the SEI, where electrons are able to reduce FDMA, it is more favorable to form Li_x_N, while the poor electronic conductivity of the SEI makes that only the first decomposition products form at the outer part of the SEI.

### Evolution of Li-metal density during plating and stripping

**Fig. 5:**
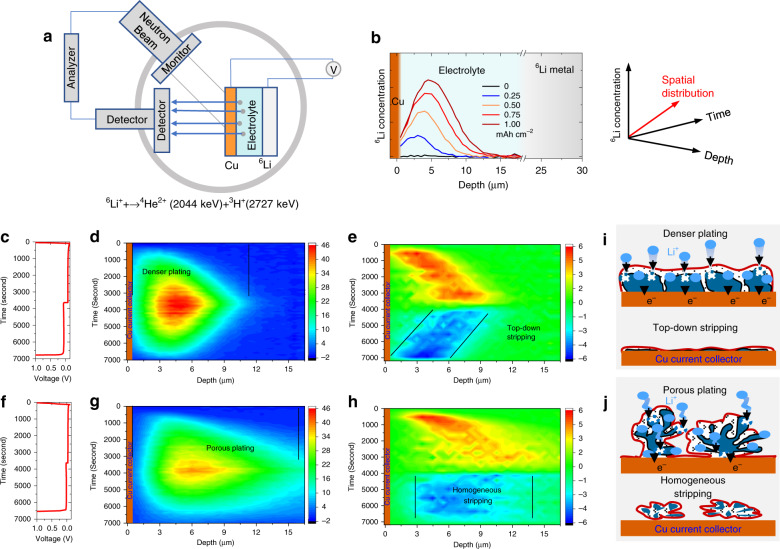
Li^+^ plating/stripping by *operando* neutron depth profile. **a** The schematic *operando* neutron depth profile (NDP) setup. **b** Depth profiles during Li metal plating/stripping on a Cu working electrode cycled at a current density of 1 mA cm^–2^ to a capacity of 1 mAh cm^–2^ of **c** 1 M LiTFSI-FEC/FDMA and **f** 1 M LiPF_6_-EC/DMC electrolytes. Evolution of Li^+^ plating/stripping density vs. time from *operando* NDP during the first cycle at 1 mAh cm^–2^ in Li||Cu pouch cells for **d** 1 M LiTFSI-FEC/FDMA and **g** 1 M LiPF_6_-EC/DMC electrolytes. The depth is calculated starting from the inner surface of the Cu current collector. Evolution of Li^+^ plating/stripping activity for **e** 1 M LiTFSI-FEC/FDMA and **h** 1 M LiPF_6_-EC/DMC electrolytes, which is obtained from the change in Li^+^ density upon each time step of **d** and **g**, respectively. The color scale in NDP measurements indicates the local Li concentration in mol/liter. **i** and **j** Schematic representation of the plating/stripping mechanism in the two electrolytes.

*Operando* neutron depth profiling (NDP) allows non-invasive monitoring of the spatial and temporal distribution of Li during electrochemical plating/stripping, providing direct visualization of the Li^+^ transport and irreversible processes^49,50^. The schematic setup of the *operando* NDP measurement and the corresponding principle are shown in Fig. 5a. Through the neutron capture reaction of the ^6^Li^+^ isotope (natural abundance 7.5%), two charged particles with a well-defined energy are produced, ^4^He^2+^ (E_k_ = 2044 keV) and ^3^H^+^ (E_k_ = 2727 keV). Due to the lower charge of the ^3^H^+^ particles, these are able to exit the pouch cells, whereas the stopping power towards ^4^He^2+^ is too large to penetrate the current collector^49^. Based on calculating the energy loss of the ^3^H^+^ particles, measured in the detector positioned perpendicular to the electrode, the ^6^Li^+^ density depth profile can be monitored during battery operation as shown in Fig. 5b. With a sub-micron depth resolution, and time resolution in the order of minutes, *Operando* NDP provides the unique opportunity to monitor Li plating/stripping quantitatively, under realistic electrochemical conditions.

Cycled at a current density of 1 mA cm^–2^ with a capacity of 1 mAh cm^–2^, the evolution of Li^+^ plating/stripping density vs. time are obtained from *operando* NDP during the first cycle in Li||Cu pouch cells for the 1 M LiTFSI-FEC/FDMA (Fig. 5c, d) and 1 M LiPF_6_-EC/DMC (Fig. 5f, g) electrolytes, respectively. As demonstrated by Fig. 5d, a denser and thinner Li deposition is observed for the amide-based electrolyte. In comparison, a much less compact and thicker Li deposit is observed for the EC-based electrolyte, extending to 18 μm into the electrolyte. These results are in good agreement with the ex situ SEM and in situ optical microscopy measurements in Fig. 3. It’s worth mentioning that the NDP measurements provide a more precise picture of the Li deposition as compared to the in situ optical microscopy, because for the latter there is a large space between the Li-metal and separator to get a clear observation of the Li-deposits, which is different to the practical cell. In the EC-based electrolyte, the Li density profile in Fig. 5g demonstrates a clear asymmetry comparing plating and stripping, consistent with the optical analysis. This phenomenon is more clearly visualized in the time derivative of the Li density, shown in Fig. 5h, representing the depth-resolved plating/stripping activity. The Li-metal stripping in the EC electrolyte is homogeneously distributed over the thickness of the deposit, whereas the stripping activity in the amide electrolyte moves back symmetrically, as compared to the plating, to the current collector. Homogeneous stripping will result in a porous Li-metal morphology, which promotes the formation of a high surface area and of Li-metal domains that are disconnected from the Cu current collector, thus resulting in the formation of “dead” Li. In contrast, stripping from the top of the deposit, as observed for the amide electrolyte in Fig. 5e and Supplementary Fig. 14, suggests a much more reversible stripping mechanism. Also, at higher and lower current densities, 0.5 and 5 mA cm^−2^, the evolution of the Li density still keeps reversible during plating and stripping using the amide electrolyte as shown in Supplementary Fig. 15 and Supplementary Fig. 16, respectively. The ability of NDP to quantitatively monitor the amount of Li metal on the current collector allows us to determine the Li efficiency, defined as the ratio between the stripped and the plated Li mass. This provides complementary information to the electron efficiency (Coulombic efficiency) where the difference quantifies the amount of irreversible reactions that do not involve Li-ion transfer, such as direct electrolyte reduction and chemical dissolution of Li from the SEI. Comparison between the Li-mass evolution in different electrolytes is shown in Supplementary Fig. 17, the amide electrolyte shows a better Li-efficiency and a higher Li mass after plating, indicating a lower amount of parasitic reduction reactions.

The origin of the top-down stripping and/or more reversible plating/stripping can be rationalized by the schematic representation in Fig. 5i, j. According to the XPS results, the SEI formed in the as-prepared electrolyte contains more ionic conductive components, which can be responsible for the denser Li metal deposits observed with *operando* NDP, as well as with SEM (Fig. 3b) and in situ optical microscopy measurements (Fig. 3d). Compared to the more porous Li metal/SEI morphology in the conventional electrolyte, compact Li metal deposition will allow more facile electron transport from the surface of the deposits to the Cu current collector, which will facilitate stripping from the top. In contrast, a more porous deposition will compromise electron transport and facilitate ion transport, resulting in relatively homogenous stripping. The consequential formation of isolated regions of Li metal, alongside a larger amount of decomposition reactions due to the porous morphology, will lower the Coulombic efficiency and hence shortening the cycle life.

### Electrochemical performance of full cells

**Fig. 6:**
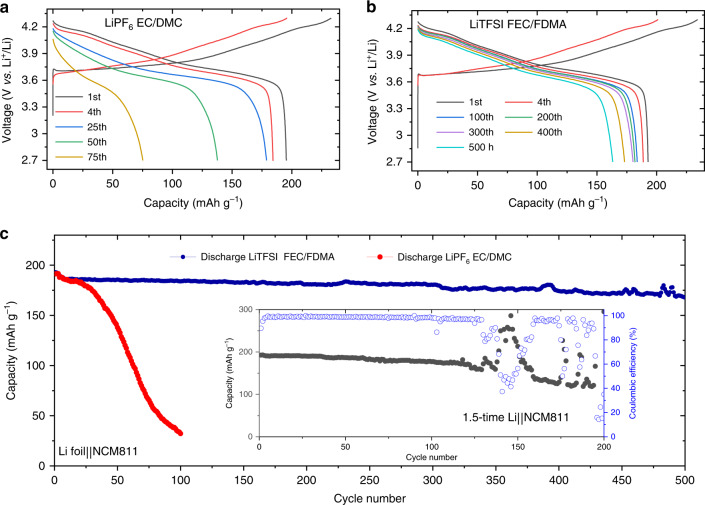
Electrochemical behavior of full cells using NCM811 as cathode. **a**, **b** Galvanostatic charge–discharge curves of Li||NCM811 cells in 1 M LiPF_6_-EC/DMC and 1 M LiTFSI-FEC/FDMA. **c** Cycling stability of Li||NCM811 cells at rate of 0.25 C with first three cycles at 0.1 C. The inset shows the cycling history of the Li||NCM811 full cells with negative to positive electrode capacity ratio of 1.5.

Ni-rich compounds are considered to be promising cathode materials for LIBs due to their large capacity, which in combination with a Li-metal anode results in a very high energy density^19,51–53^. In this context, NCM811 with a high active mass loading of 3.5 mAh cm^−2^ is used to test the performance of the prepared amide electrolyte. The high cathode mass loading aggravates the side reactions and requires a high efficiency of the Li-metal utilization^54^. Both Li||NCM811 cells, employing the different electrolytes, deliver similar initial charge and discharge voltage profiles, resulting in a specific capacity of 195 mAh g^−1^, shown in Fig. 6a, b. Upon subsequent cycling, the full cells with the amide electrolyte present much better cycling stability, retaining >88% of its initial capacity after 500 cycles as shown in Fig. 6c, and a high average Coulombic efficiency as shown in Supplementary Fig. 18. In comparison, the battery with the EC-based electrolyte rapidly decays to ~30% after 75 cycles as shown in Fig. 6a, c. Evaluation of the C-rate charging-discharging performance of the full cells, see Supplementary Fig. 19, demonstrates better rate capabilities for the amide-based electrolyte. This is in line with the smaller impedance observed, shown in Supplementary Fig. 20, which demonstrates the good compatibility of the amide electrolyte in full Li-metal cells. Furthermore, the full cycling history of a Li||NCM811 full cell with a restricted Li capacity is evaluated in Fig. 6c, where the Li-metal capacity on the Cu current exceeds the cathode capacity (~3.5 mAh cm^−2^) by a factor of 1.5. Under these demanding conditions stable cycling performance is achieved as >92% of the capacity is retained for 100 cycles. In addition, when FDMA is added to the conventional electrolyte system, as shown in Supplementary Fig. 21, the Li||NCM811 full cell using 1 M LiPF_6_-EC/DMC/FDMA (volume ratio 1:1:1) exhibits improved cycling stability compared with using 1 M LiPF_6_-EC/DMC.

### Interface between cathode and electrolyte

The interface stability between cathode and electrolyte is further investigated by SEM and TEM. After 50 cycles in full cells, the morphology of the cathode materials is well preserved in the amide electrolyte, while obvious cracks and pulverization of the cathode are observed for the secondary particles in the EC-based electrolyte, see Supplementary Figs. 22 and 23. This indicates that the as-prepared amide electrolyte can suppress the structural degradation of NCM cathode materials. More evidence is provided by the transmission electron microscope (TEM) characterization. When cycled in the EC-based electrolyte, severe surface damage on the cycled cathodes is observed in Supplementary Fig. 24a, indicated by the yellow dashed lines. The corresponding fast Fourier transforms (FFT) patterns of the near-surface regions, up to 12 nm in depth, demonstrate the phase transition from the original layered structure to the rock-salt phase at the surface. In contrast, the surface layer on the particle cycled in the amide electrolyte is much thinner, ~4 nm, and more uniform. Moreover, it is mainly composed of amorphous cathode electrolyte interface (CEI) components as shown in Supplementary Fig. 24b. This indicates that this amide electrolyte effectively protects the cathode surface. Energy dispersive spectroscopy mapping clearly shows the presence of N- and F-containing components in the CEI layer, Supplementary Fig. 24c, explaining the decreased resistance and the improved electrochemical stability.

Furthermore, the chemical composition of CEI layers is investigated by XPS, results of which are shown in Supplementary Figs. 25–30. In the EC-based electrolyte, four peaks in the O 1s spectrum, located at about 530.5, 532.1, 533.4, and 534.3 eV, can be indexed as M–O (metal oxide from the cathode bulk), C=O, C–O, and Poly(CO_3_), respectively^55^. The appearance of the M–O species indicates that the CEI layer is cracked and non-uniform, exposing the cathode surface. In the O 1s and F 1s spectra of the amide electrolyte, a new peak indicates the presence of –CF_3_, which may originate from either the LiTFSI salt or FDMA. In addition, the CEI formed in the amide electrolyte is richer in LiF, as demonstrated by Supplementary Fig. 26. LiF has a large oxidative stability and excellent mechanical stability, which can be held responsible for the high-voltage stability of the CEI layer^17,19^. The N-containing species are also observed in the CEI layer of the amide electrolyte, which provides the required Li transport through the CEI layer. These results demonstrate that the amide electrolyte, through the synergy between both the FEC and FDMA, also provides a homogeneous and stable CEI protective layer, leading to the improved electrochemical performance of the full cells.

## Discussion

In this work, a new electrolyte is prepared that enables highly reversible and stable cycling of lithium metal batteries. The as-prepared electrolyte, combining FDMA with FEC as a cosolvent, shows significantly enhanced electrochemical performance of Li-metal anodes in combination with high-loading Ni-rich batteries as compared to conventional carbonate-based electrolytes. The rationale behind this is the relatively low LUMO level of FDMA, which initiates decomposition into ion-conductive N-containing components. In combination with the highly stable F-components in both SEI and CEI layers, Li dendrites and porous Li-metal morphologies are reduced and both anode and cathode degradation are effectively prevented. Monitoring the Li-metal deposition with in situ optical microscopy and *operando* NDP shows that the amide-based electrolyte is responsible for denser Li deposition, which induces Li^+^ stripping from the top of the deposit towards the current collector. As opposed to homogeneous stripping observed in carbonate electrolytes, the top-down stripping lowers the chance to form inactive regions of Li metal and contributes to less porous morphologies, hence suppressing irreversible reactions, which is responsible for the large Coulombic efficiency. The understanding of the relationship between the interface chemistry and the Li^+^ plating/stripping mechanism in this promising electrolyte, opens up new opportunities to improve the reversibility of lithium metal batteries.

## Methods

### Materials

Battery grade ethylene carbonate (EC, Sigma-Aldrich) and dimethyl carbonate (DMC, Novolyte) (1:1 volume ratio), lithium hexafluorophosphate (LiPF_6_, Sigma-Aldrich), lithium bis(trifluoromethanesulfonyl)imide (LiTFSI, Sigma-Aldrich) and fluoroethylene carbonate (FEC, Sigma-Aldrich) were used and stored in a Ar glovebox with measured levels of oxygen and water contents below 1.0 ppm. 2,2,2-Trifluoro-*N, N*-dimethylacetamide (FDMA, >98.0%(GC)), was purchased from Tokyo Chemical Industry Co., Ltd with further dehydration. Single glass fiber (Whatman, GF/A) separator is used for all cells. The LiNi_0.8_Co_0.1_Mn_0.1_O_2_ (NCM811) electrode contains 94.5 wt.% active material, 3 wt.% conductive carbon, and 2.5 wt.% polyvinylidene fluoride binder in *N*-methyl-2-pyrrolidone coated on an aluminum (Al) current collector foil with an active material mass loading of 18 mg cm^−2^. The electrodes were punched into disks with a diameter of 12 mm and dried under vacuum for 12 h at 80 °C before use.

### Electrochemical measurements

Li||Cu, Li||Li and Li||NCM811 cells were assembled in standard 2032 coin-type cells using GF/A separator and about 70~120 μL electrolyte in the Ar-filled glove box with oxygen and water contents below 1.0 ppm. The cells were tested on a Maccor 4400 cycling system. The copper foil for Li||Cu cells were pouched into disks with diameter of 16 mm as working electrodes, while Li metal was used as the reference and counter electrode. During each cycle, a designed amount of Li was deposited on the Cu foil at a specific current density and then stripped until the potential reached 1.0 V vs. Li^+^/Li. Symmetric Li||Li cells were also assembled to study the cycling stability under different current densities in different electrolytes. Li||NCM811 full cells using Li foil as the anode were cycled in galvanostatic mode with a voltage range of 2.7–4.3 V. The electrolyte weight to cathode capacity ratio in 1 M LiTFSI-FEC/FDMA is around 30 g Ah^−1^, and that in 1 M LiPF_6_- EC/DMC is around 25 g Ah^−1^. Linear sweep voltammetry (LSV) was conducted with stainless steel served as working electrode and Li metal foil as both counter and reference electrodes. The LSV measurements were carried out on an Autolab (PGSTAT302N) with a scan rate of 0.1 mV s^−1^ from open-circuit voltage to 6 V vs. Li^+^/Li. The electrochemical impedance spectra (EIS) of the symmetric cells and full cells were collected on an Autolab (PGSTAT302N) in the frequency range of 0.1 Hz–1 MHz with a potential amplitude of 10 mV. The Li metal anode for the Li||NCM811 full cell with a restricted Li capacity is prepared by electrochemical plating. A Li||Cu cell using 1 M LiTFSI-FEC/FDMA was discharged at 1 mA cm^−2^ to a capacity of 1.5 times that of the NCM811 electrode, subsequently, the cell was disassembled to obtain the thin Li metal anode.

### Characterizations

The morphology of electrodes cycled with different electrolyte were characterized by SEM (SEM, Hitachi SU-4800). Before the sample was mounted on the sample holder, the deposited Li metal on the Cu foil and the NCM811 electrode after 50 cycles were disassembled and washed 3 times with anhydrous DMC to remove the residual electrolyte and dried in the glovebox to remove the solvent residual. To characterize the detailed surface morphology of the cathode powder after cycling, transmission electron microscopy (TEM, FEI Tecnai G2 spirit) was used.

The XPS testing was carried out to analyze the composition of both anode and NCM811 cathode after cycling, the cells were disassembled in an Ar-filled glove box. The electrodes were rinsed with DMC solvent 3 times to remove residual electrolyte, followed by dried under vacuum condition for 12 h at room temperature to remove the solvent residual. The element composition on the surface of the electrodes was analyzed by X-ray photoelectron spectroscopy (XPS, PHI 5000 VersaProbe II) using monochromatic Al K(alpha) X-ray source calibrated with respect to carbon (284.8 eV). Peak fitting was performed using MultiPak software.

Symmetric cells were assembled in a special designed optical cell for in situ observation. The assembly process were performed in an Argon-filled glovebox with water and oxygen content below 1.0 ppm. Li foils and glass fiber separator were placed into the optical cell vertically. After the electrolyte was injected, the whole cell was transferred to the optical microscope (Olympus BXFM) located in the glovebox, and was cycled using a CT2001A tester (LAND Electronic Co. Ltd., Wuhan). In situ observations were carried out in the glovebox at ambient temperature in dark field shooting by a fitted charge-coupled device (CCD) camera on the microscope.

*Operando* neutron depth profiling (NDP) was performed on the thermal neutron beamline at the Reactor Institute Delft, the Netherlands. The specially designed air-tight Li||Cu pouch cell with a window for the NDP experiments was assembled using ^6^Li metal (95% wt.% ^6^Li and 5% wt.% ^7^Li), Glass fiber disks (GF/A, Whatman) next to a 25 μm PE (Celgard), Cu foil (~10 μm, as working electrode and the window), and 150 μl electrolyte. The pouch cell was fixed inside the stainless-steel chamber filled by a 1 bar He atmosphere, and the whole box was fixed in to an Al vacuum chamber, where the experiments were performed. The cells are positioned at an angle of 30° towards the incident neutron beam and parallel to the Canberra PIPS detector placed perpendicular to the battery electrodes at 4.5 cm from the pouch cells in order to measure the energy of the emitted ^3^H particles. Galvanostatic cycling was performed using a Maccor 4000 cycling system by plating Li onto the Cu working electrode at 1 mA cm^−2^ to a total capacity of 1 mAh cm^−2^ followed by stripping to a cut-off voltage of 1.0 V vs. Li^+^/Li. SRIM was used to calibrate the stopping power of the materials for converting the triton (^3^H) energy loss and intensity to the Li depth and Li density^56^. For the calculation of the stopping power and the depth calibration, we refer to previous work^49,57^.

### Calculations

Quantum chemical calculations were conducted using density functional theory (DFT) method with Becke’s three parameters (B3) exchange functional in Lee-Yang-Parr (LYP) nonlocal correlation functional (B3LYP)^58,59^. All the geometry optimizations were proceeded with B3LYP/6-31+G(d,p) level. The energy calculations were performed at B3LYP/6-311+ +G(3df,3dp) level for more accurate calculation. All DFT calculations were performed by using the Gaussian 09 program package^60^.

## Supplementary information

Supplementary Information

Description of Additional Supplementary Files

Supplementary Movie 1

Supplementary Movie 2

## Data Availability

The data that support the findings within this paper are available from the corresponding author on request.
