# Supplementary material for: Interface chemistry of an amide electrolyte for highly reversible lithium metal batteries
Source: Nat Commun. 2020 Aug 21;11:4188. doi: 10.1038/s41467-020-17976-x (PMC7442789; doi:10.1038/s41467-020-17976-x)
Supplement: Supplementary file 1 — Supplementary Information [file 41467_2020_17976_MOESM1_ESM.pdf]

Supplementary information

Interface chemistry of amide electrolyte for highly reversible lithium metal batteries

Wang et al.

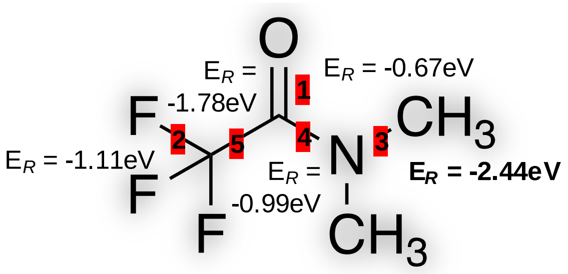

Possible mechanisms:

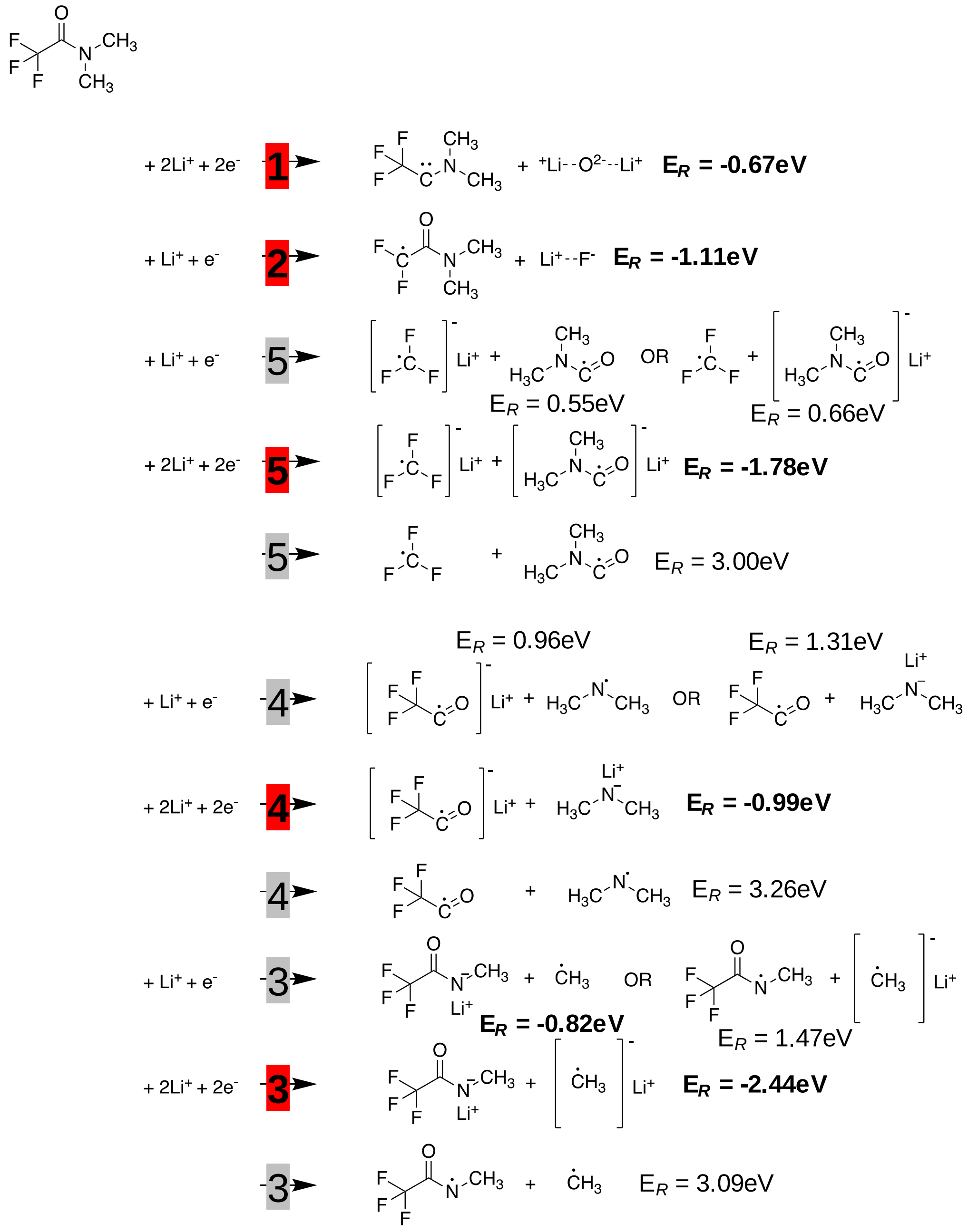

Supplementary Figure 1. Possible decomposition mechanisms of FDMA.

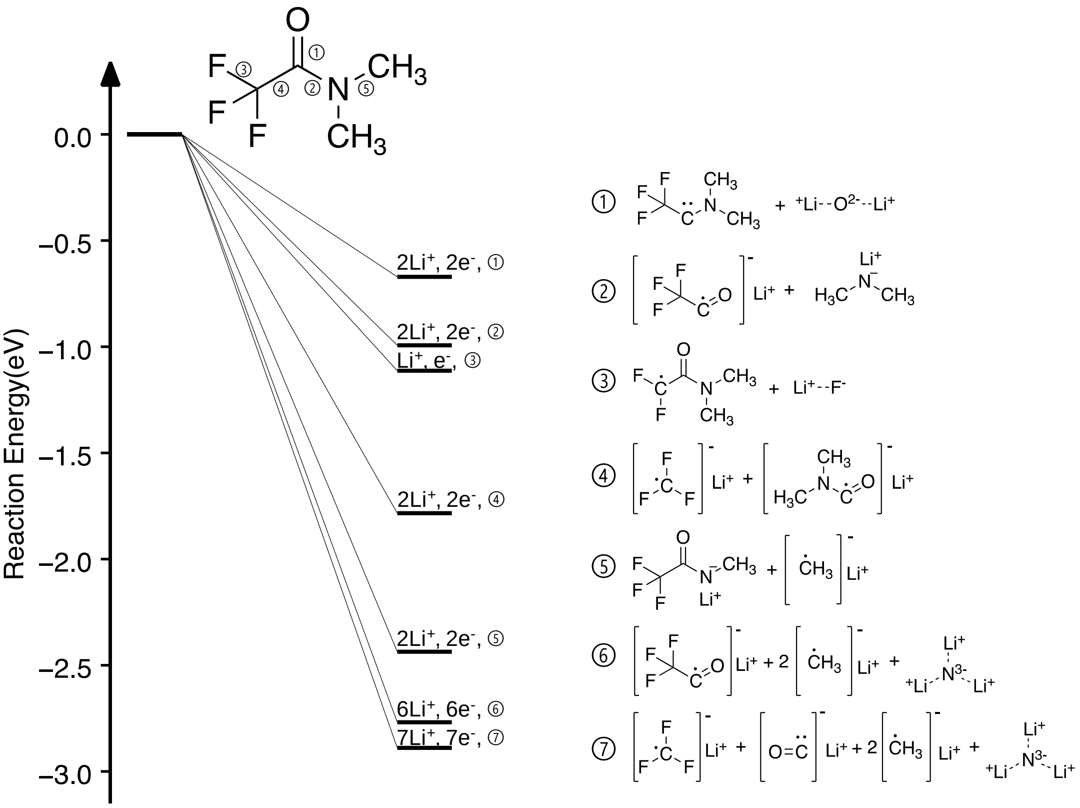

Supplementary Figure 2. Summary of the reaction energy of for attack of Li^{+} and e^{-}.

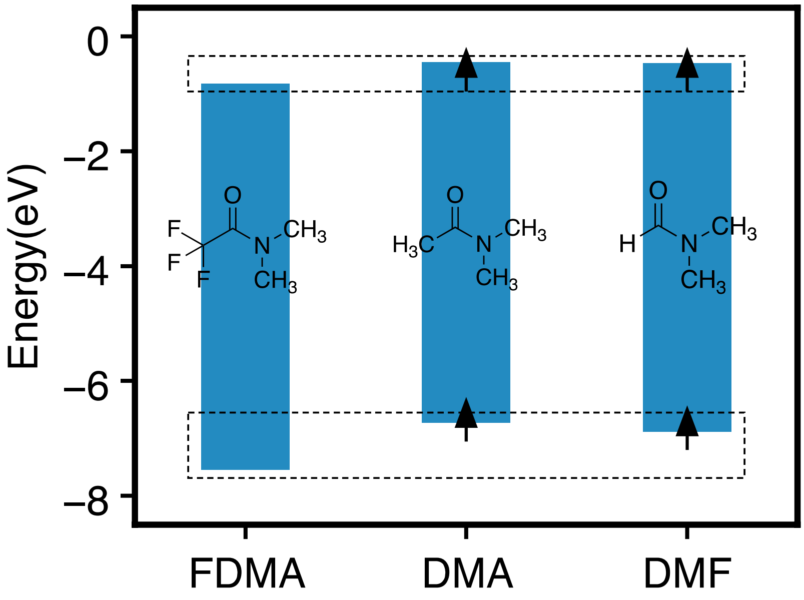

Supplementary Figure 3. Influence of F substitution in amide solvent. Comparison of highest occupied molecular orbital (HOMO)-lowest unoccupied molecular orbital (LUMO) energy levels for 2,2,2-Trifluoro-*N*, *N*-dimethylacetamide (FDMA), *N*, *N*-dimethylformamide (DMF) and dimethylacetamide (DMA).

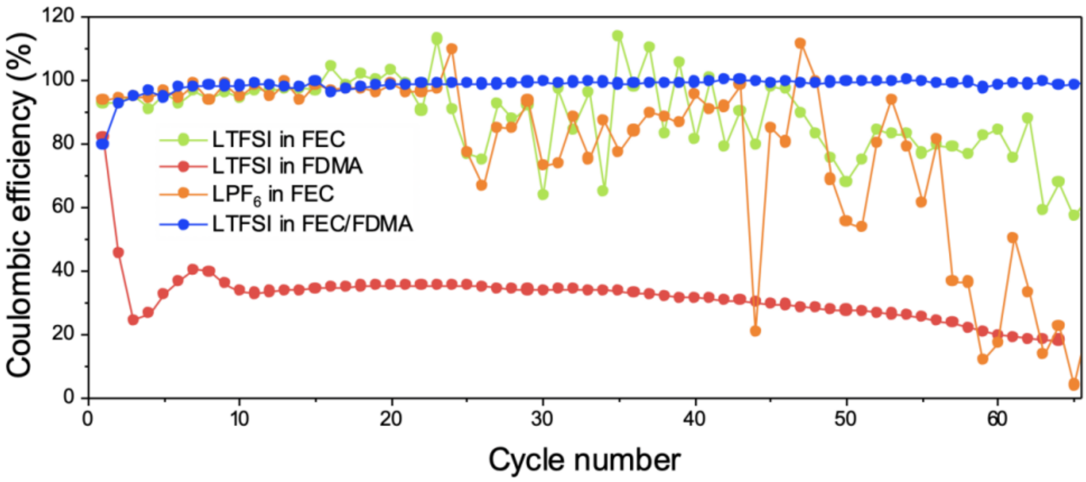

Supplementary Figure 4. Coulombic efficiency in Li||Cu cells using different electrolytes. Li was electrodeposited at 1 mA cm^{-2} to a total capacity of 1 mAh cm^{-2} followed by stripping to a cut-off voltage of 1.0 V *vs.* Li^+/Li.

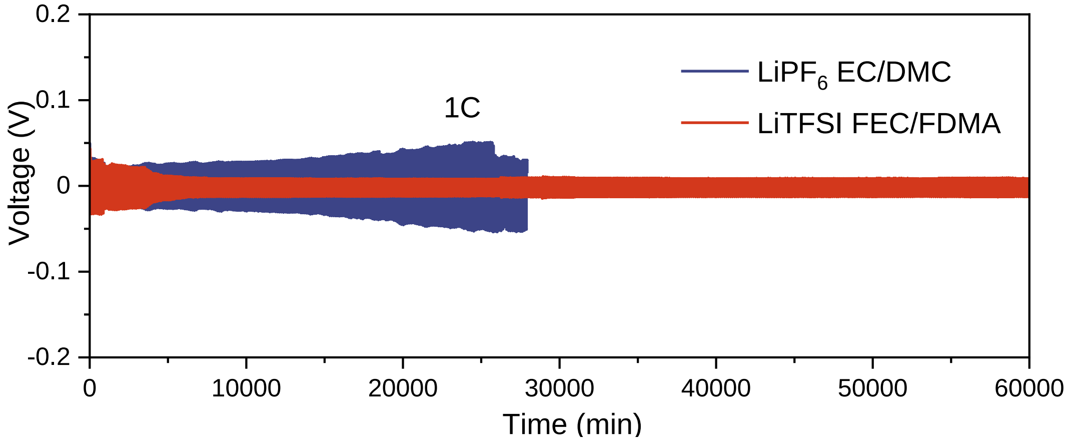

Supplementary Figure 5. The evolution of voltage profiles. The symmetric Li||Li cells cycling in different electrolytes under a current density of 1 mA cm^{-2} with each plating/stripping time of 1h.

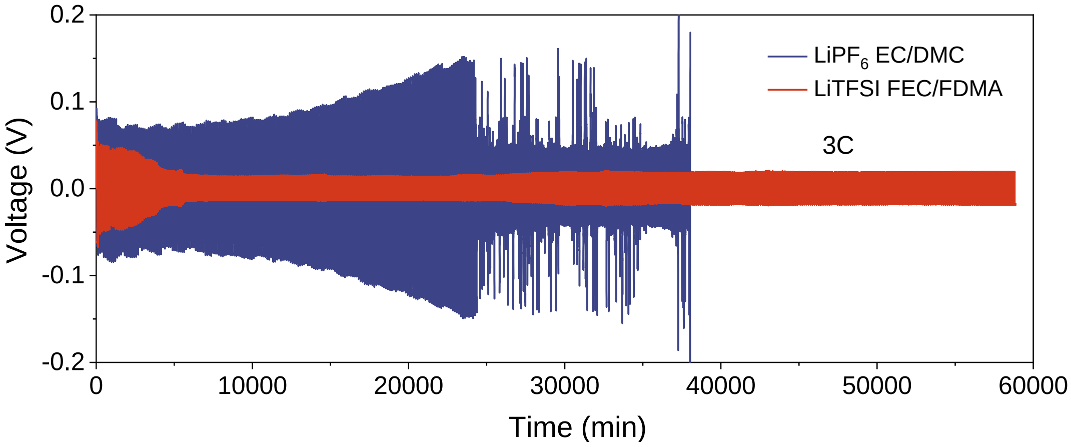

Supplementary Figure 6. The evolution of voltage profiles. The symmetric Li||Li cells cycling in different electrolytes under a current density of 3 mA cm^{-2} with each plating/stripping time of 1 h.

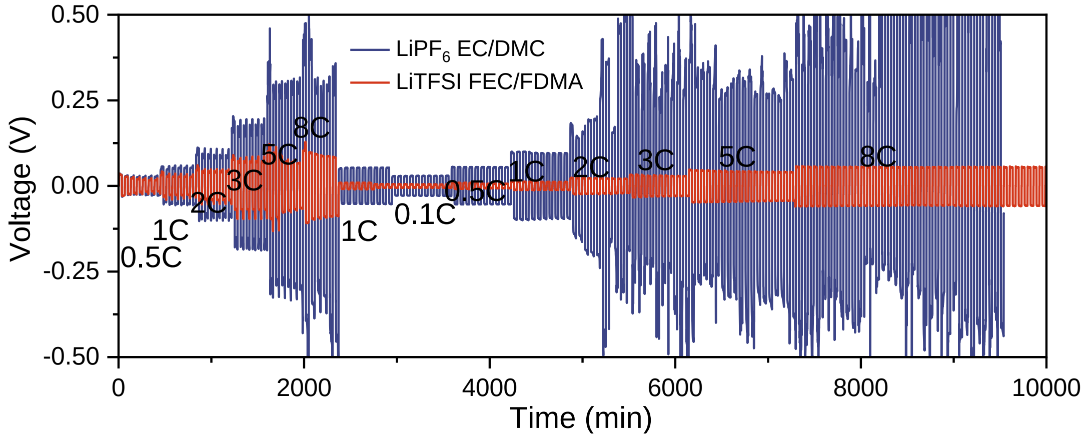

Supplementary Figure 7. Rate profile for symmetric cells with different electrolyte. Li||Li cells at current density from 0.5 to 8C, 1C=1 mA cm^{-2}.

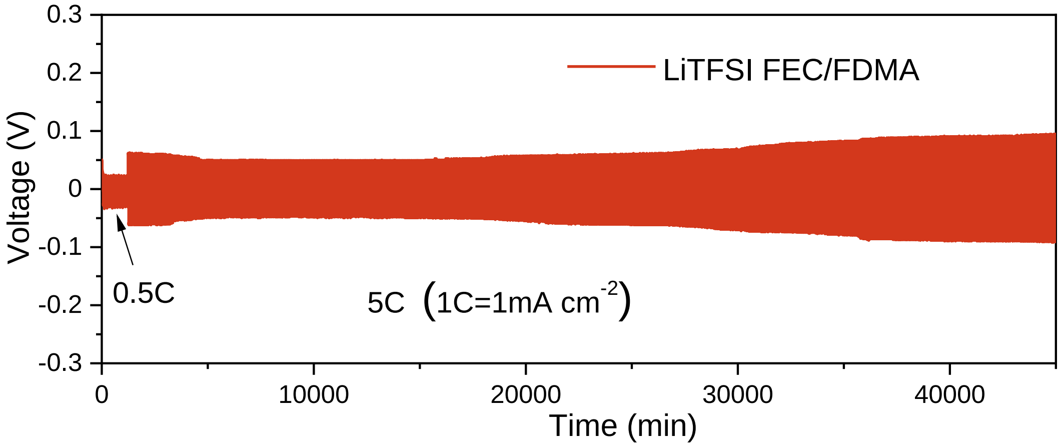

Supplementary Figure 8. The evolution of voltage profiles. The symmetric Li||Li cells over cycling in different electrolytes under a current density of 5 mA cm^{-2} with each plating/stripping time of 1h.

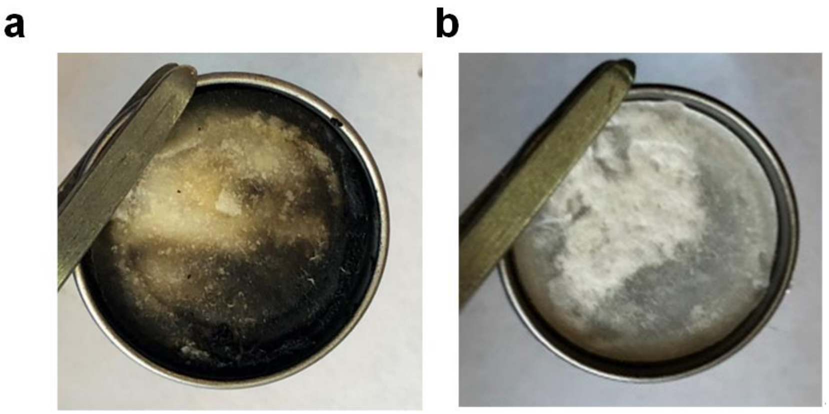

Supplementary Figure 9. Digital photos of the Li metal anodes after cycling. Li metal anodes retrieved from the symmetric Li||Li cells after 100 cycles using **a,** 1M $LiPF_6$-EC/DMC and **b,** 1M LiTFSI-FEC/FDMA.

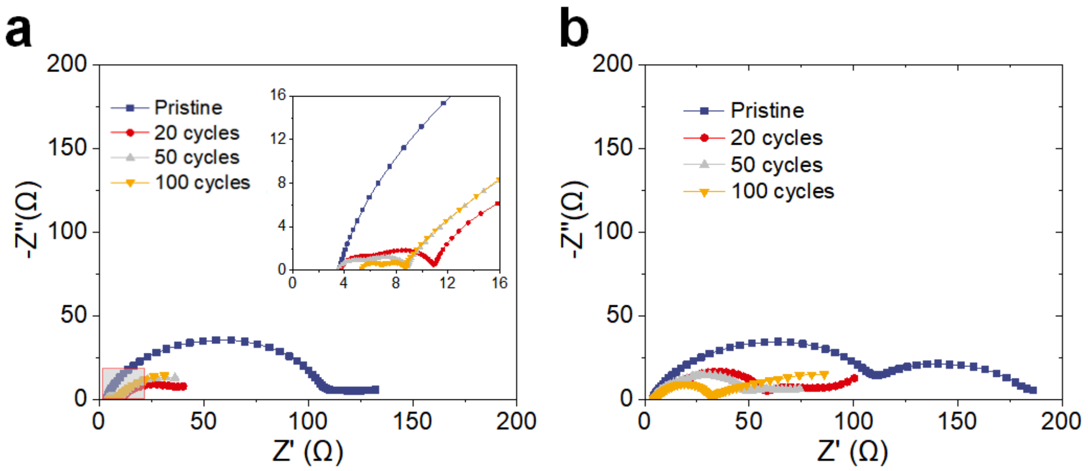

Supplementary Figure 10. Electrochemical impedance spectra of Li||Li symmetric cells. The impedance sepctras using **a,** 1 M LiTFSI-FEC/FDMA and **b,** 1 M $LiPF_6$-EC/DMC electrolytes. The insets show the enlarged view regions.

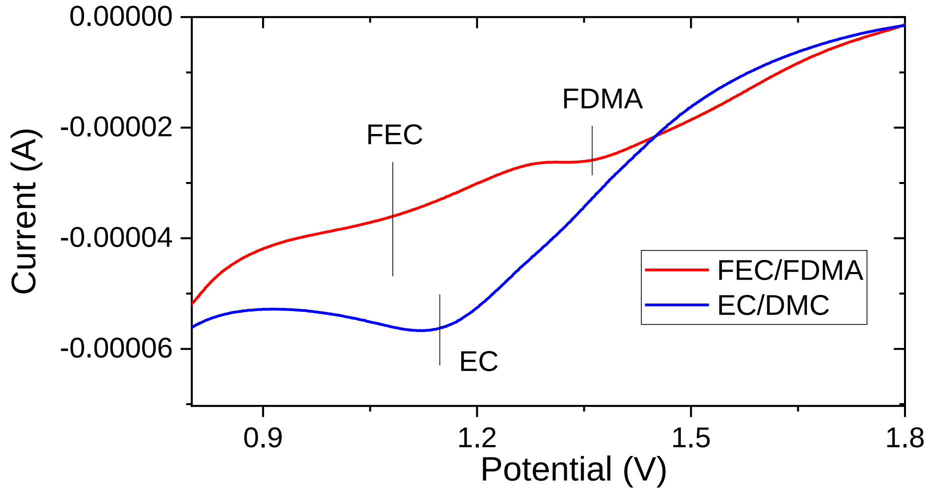

Supplementary Figure 11. Cyclic voltammetry curves of Li||Cu cells. Comparision of 1 M $LiPF_6$-EC/DMC and 1 M LiTFSI-FEC/FDMA electrolytes.

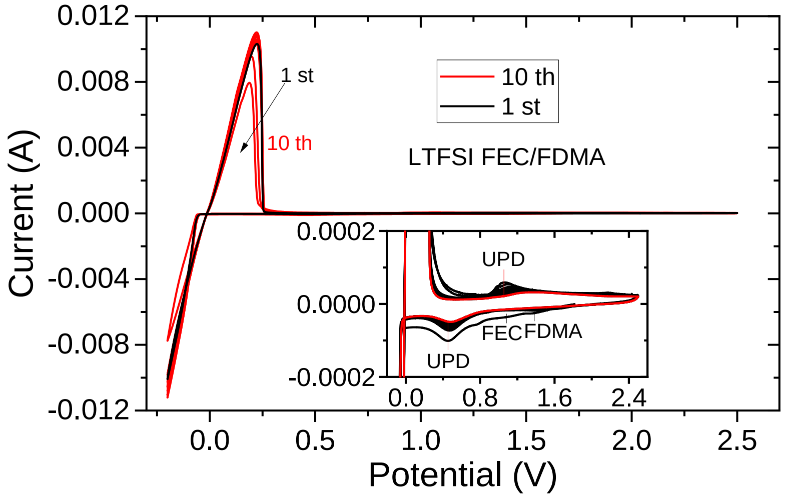

Supplementary Figure 12. Cyclic voltammetry curves of Li||Cu cells. Cyclic voltammetry curves of cell using 1 M LiTFSI-FEC/FDMA electrolyte. The insets show the enlarged view regions.

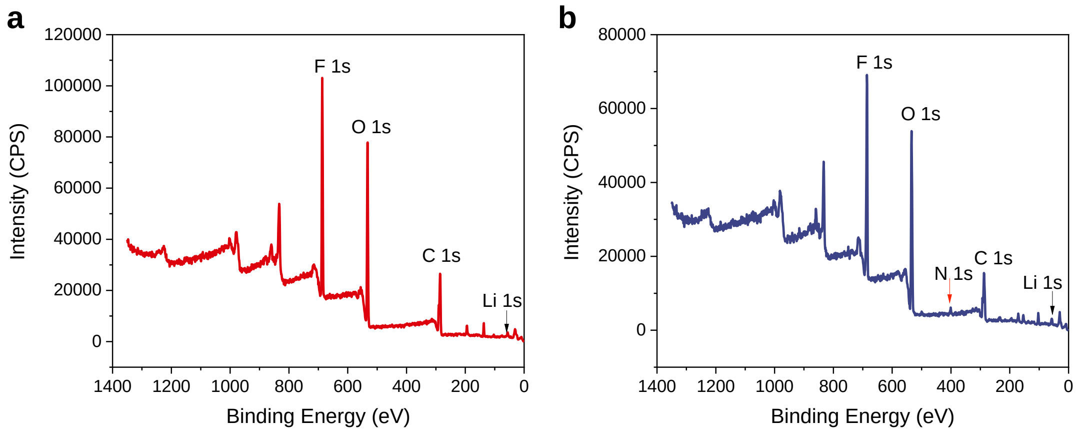

Supplementary Figure 13. The survey spectra of the XPS. XPS spectra of Li metal anodes after 50 cycles with **a,** 1M $LiPF_6$-EC/DMC and **b,** 1M LiTFSI-FEC/FDMA.

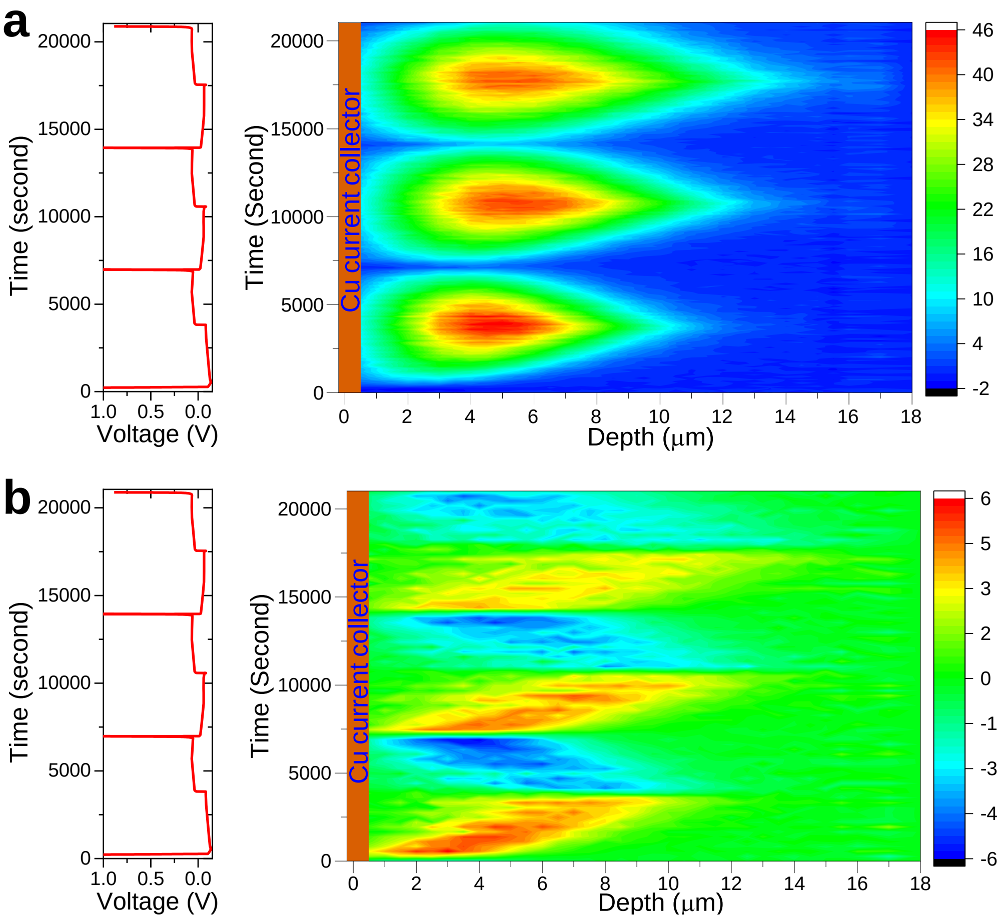

Supplementary Figure 14. The *operando* neutron depth profile results. The Li metal anode plating/stripping behavior of the first three cycles with 1M LiTFSI-FEC/FDMA under the current density of 1 mA cm^{-2}. **a** Evolution of Li^+ plating/stripping density *vs.* time. **b** Li^+ plating/stripping activity.

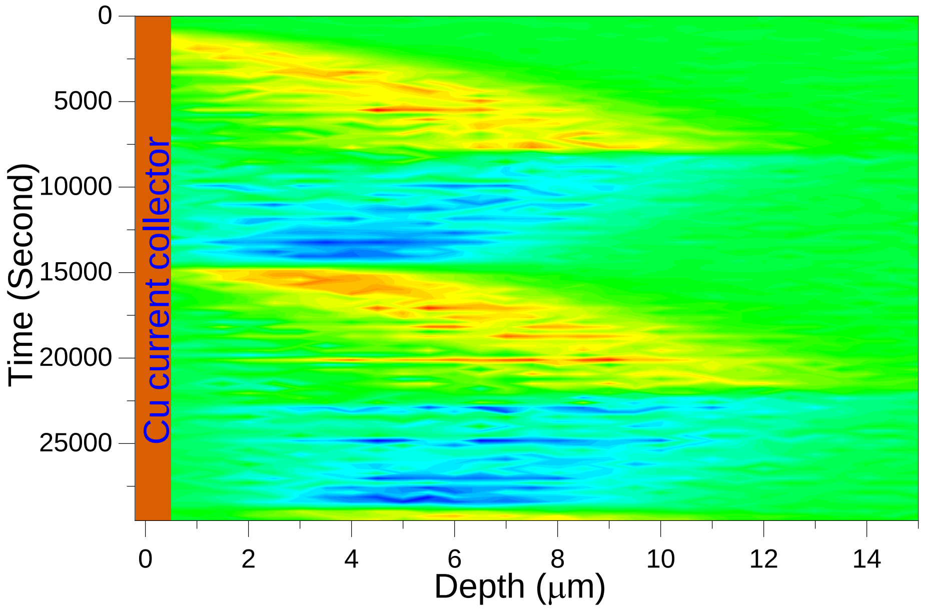

Supplementary Figure 15. The *operando* neutron depth profile of the Li metal anode. The first two cycles with 1M LiTFSI-FEC/FDMA under the current density of 0.5 mA cm^{-2}.

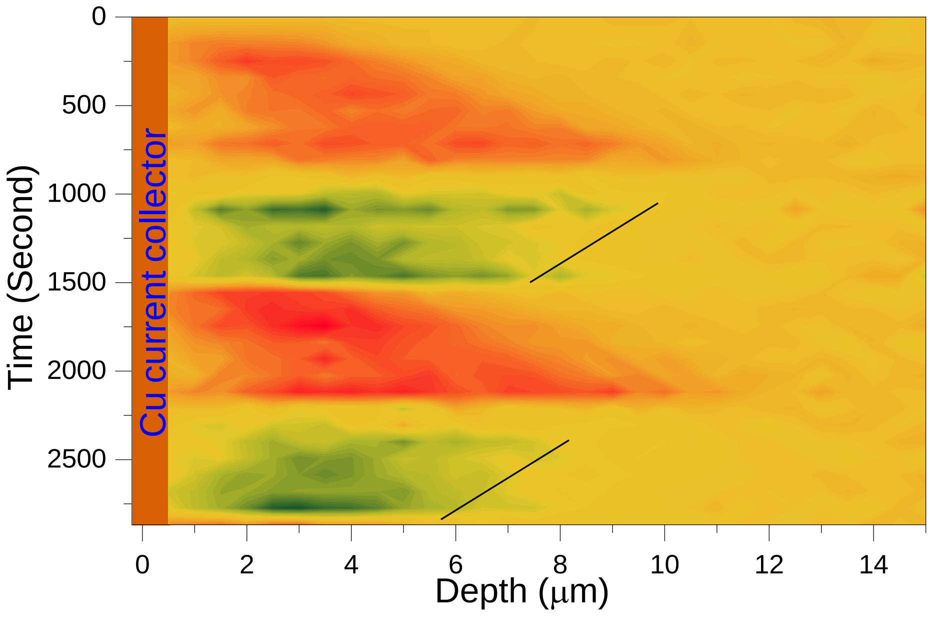

Supplementary Figure 16. The *operando* neutron depth profile of the Li metal anode. Li metal anode cycled in 1M LiTFSI-FEC/FDMA at 5 mA cm^{-2} to a capacity of 1 mAh cm^{-2}.

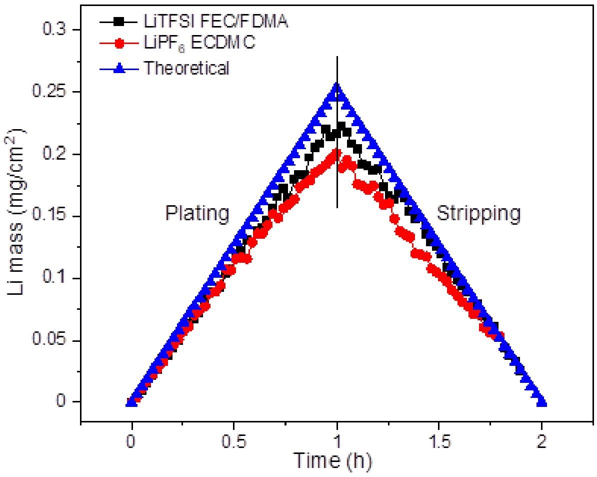

Supplementary Figure 17. Li mass evolution profile. Li mass results are obtained from theoretical calculation and integrating the *operando* neutron depth profile spectra of the batteries with different electrolytes for the plating/stripping cycle at 1 mA cm^{-2}.

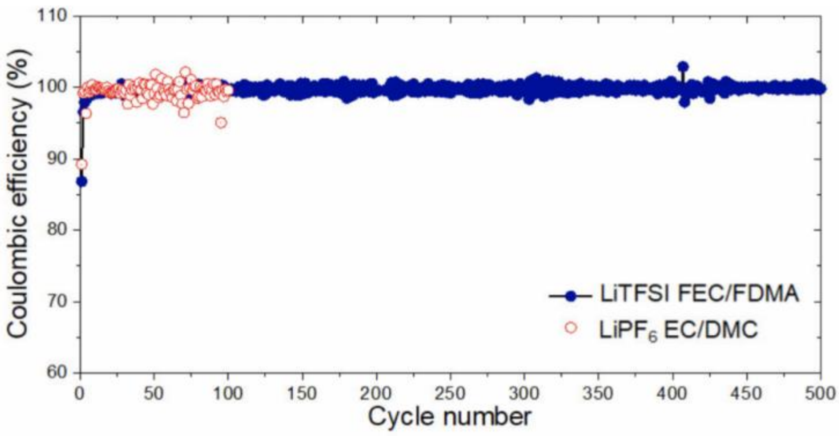

Supplementary Figure 18. Coulombic efficiency of the NMC811||Li batteries. Long cycling Coulombic efficiency of full cells using different electrolytes.

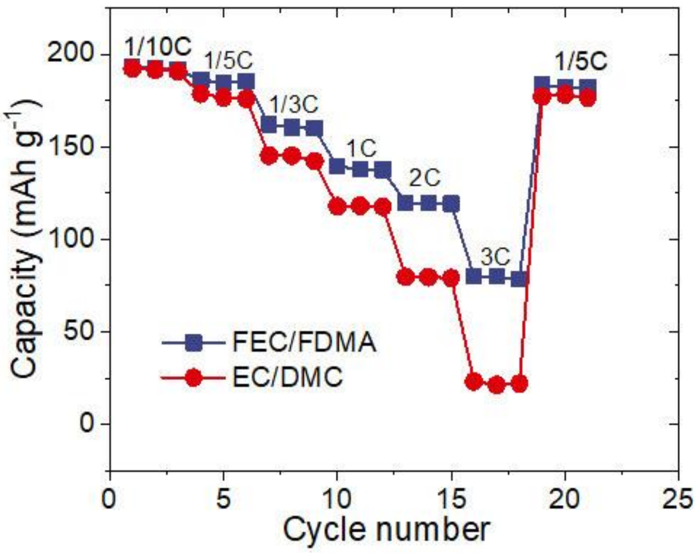

Supplementary Figure 19. Rate capability of Li||NCM full cells using various electrolytes.

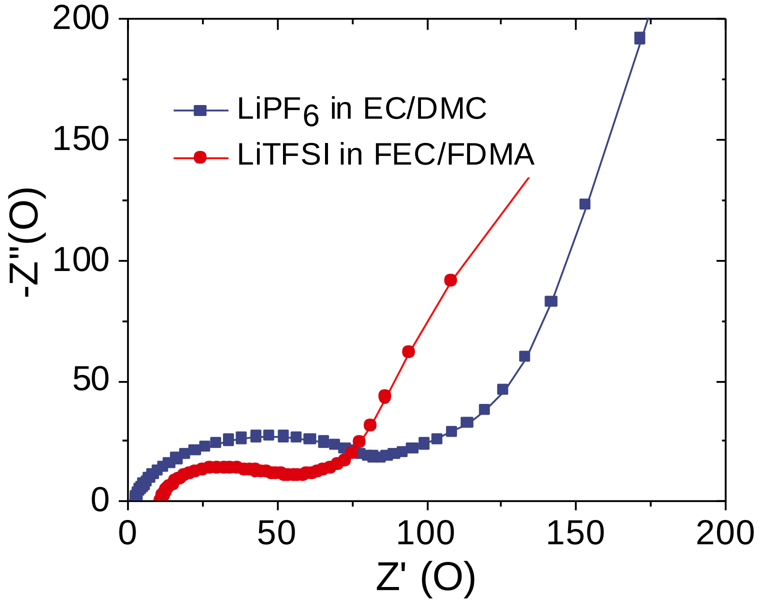

Supplementary Figure 20. Comparision of impedance spectra. Nyquist plot of Li||NCM batteries using different electrolyte before cycling.

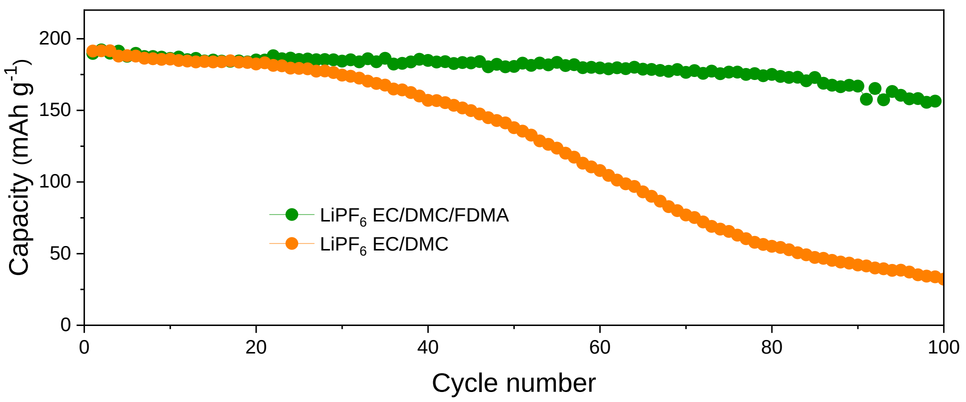

Supplementary Figure 21. Long cycling performance comparision of full cells. Cycling performance of Li||NCM811 cells using 1M $LiPF_6$-EC/DMC/FDMA (volume ratio 1:1:1) and 1M $LiPF_6$-EC/DMC at the rate of 0.25C.

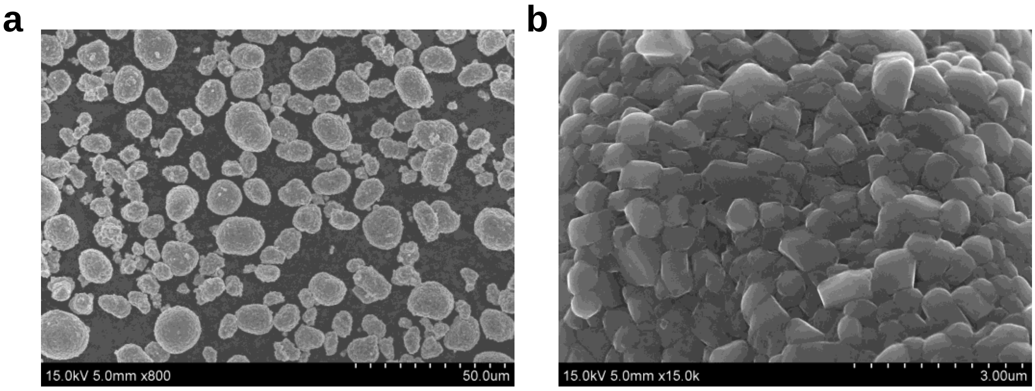

Supplementary Figure 22. Typical SEM images of uncycled NCM811cathode. a,b SEM images of NCM811 cathode under different magnifications. As can be seen from the structure of uncycled NCM811 cathode, the secondary microparticles are consisted of densely packed primary sub-microparticles with clean surface. The diameter of NCM811 particle is ranging from 5-15 μm.

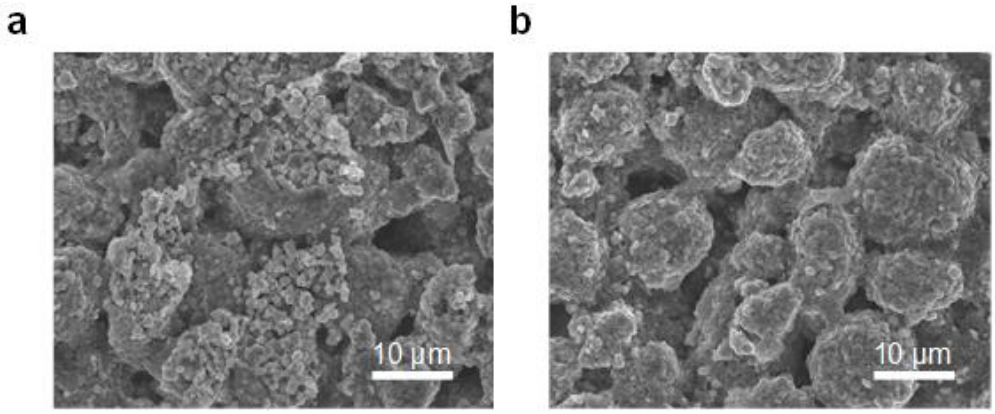

Supplementary Figure 23. Characterization of NCM811/electrolyte interface. SEM images of cycled NCM811 cathode retrieved from Li||NCM batteries using **a,** 1M $LiPF_6$-EC/DMC and **b,** 1M LiTFSI-FEC/FDMA.

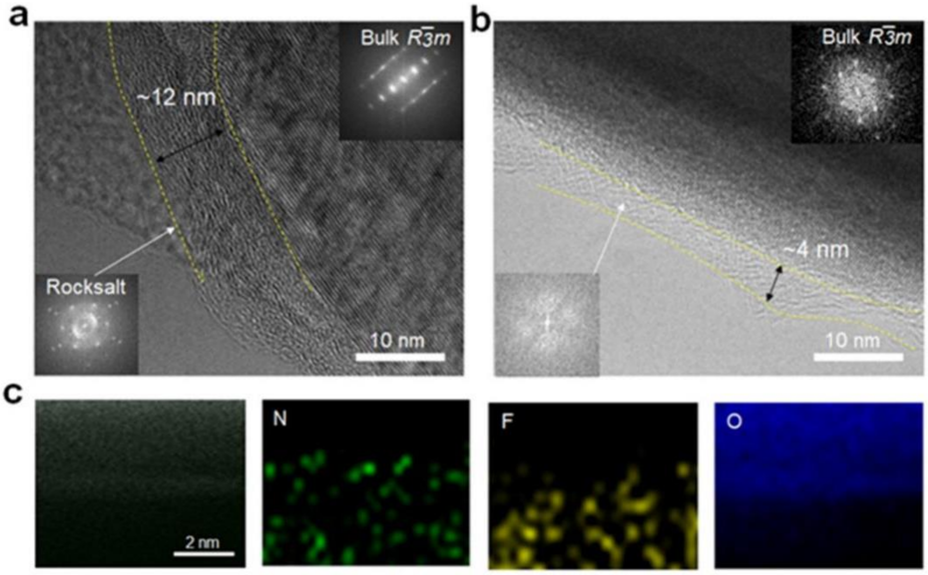

Supplementary Figure 24. Cathode-electrolyte interface in full cells after cycling. a and b, Transmission electron microscope (TEM) images of cycled NMC cathodes (50 cycles) in different electrolytes: 1M $LiPF_6$ EC/DMC and 1 M LiTFSI FEC/FDMA. Insets are the fast Fourier transform (FFT) patterns of selected regions. **c.** Energy dispersive spectroscopy mapping of selected elements.

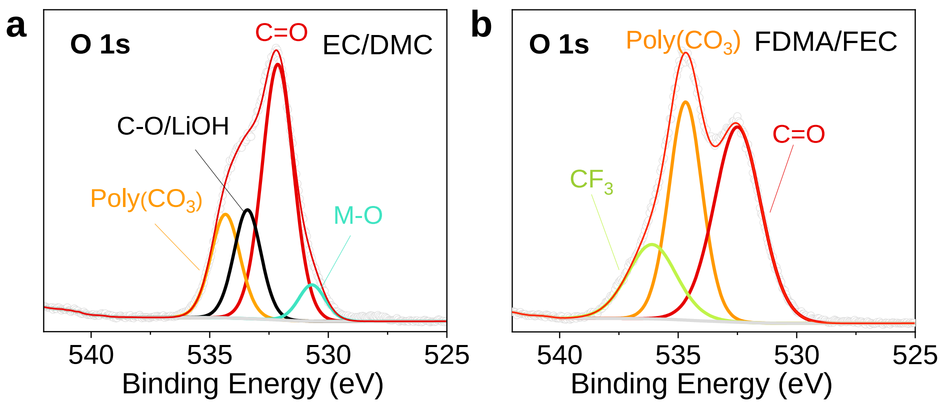

Supplementary Figure 25. O 1s spectra of XPS on NCM cathode after 50 cycles. a, 1M $LiPF_6$-EC/DMC and **b,** 1 M LiTFSI-FEC/FDMA.

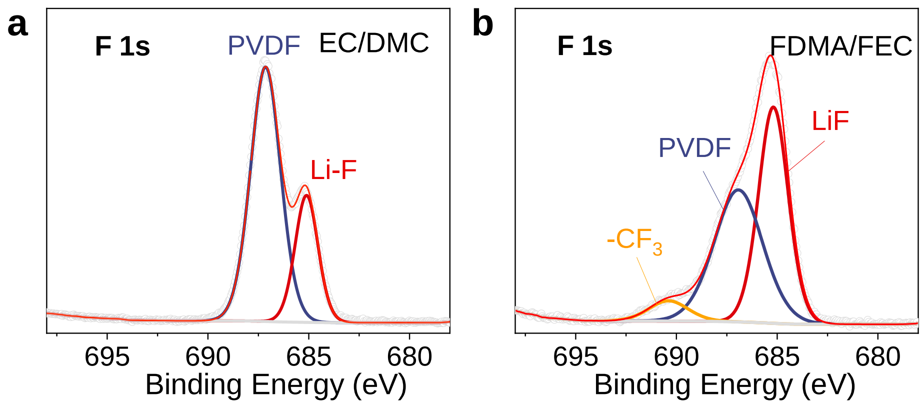

Supplementary Figure 26. F 1s spectra of XPS on NCM cathode after 50 cycles. a, 1M $LiPF_6$-EC/DMC and **b,** 1 M LiTFSI-FEC/FDMA.

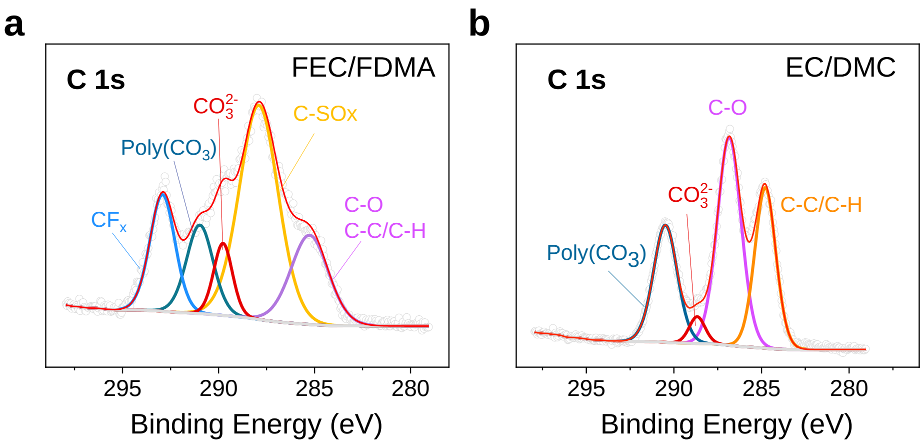

Supplementary Figure 27. C 1s spectra of XPS on NCM cathode surface after 50 cycles. a, 1M LiTFSI-FEC/FDMA and **b,** 1M $LiPF_6$-EC/DMC.

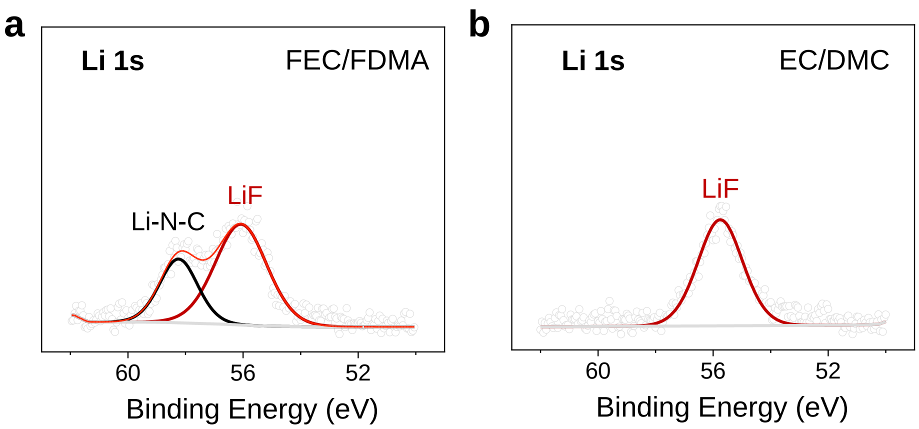

Supplementary Figure 28. Li 1s spectra of XPS on NCM cathode surface after 50 cycles. a, 1M LiTFSI-FEC/FDMA and **b,** 1M $LiPF_6$-EC/DMC.

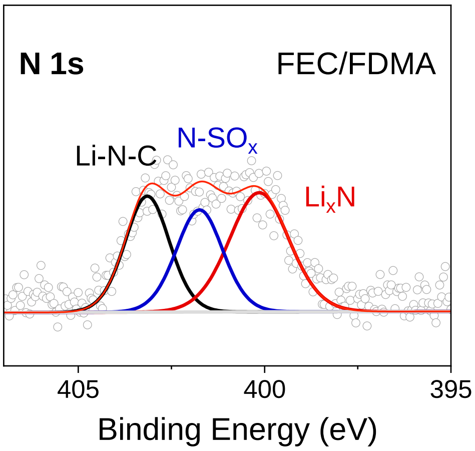

Supplementary Figure 29. N 1s spectra of XPS on NCM cathode surface after 50 cycles. 1M LiTFSI-FEC/FDMA is used as electrolyte.

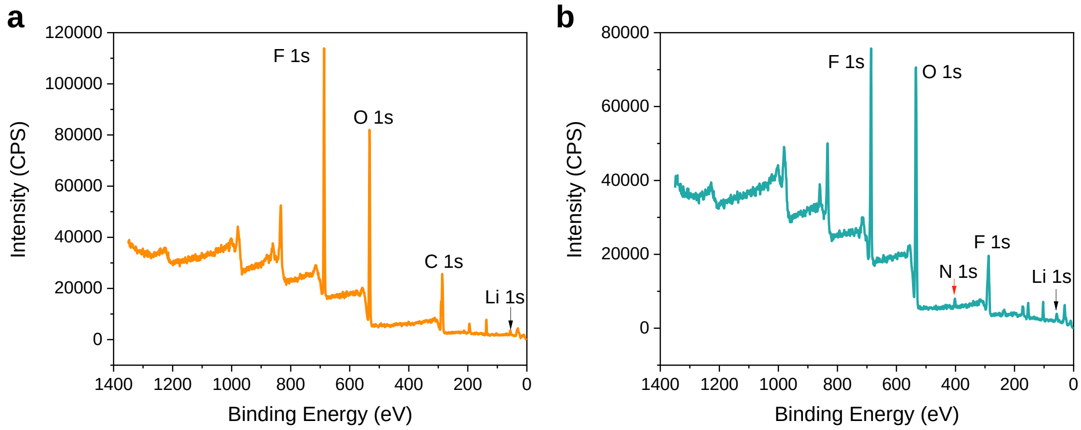

Supplementary Figure 30. The survey of the XPS spectra. XPS spectra of NCM811 electrodes after 50 cycles with **a,** 1M $LiPF_6$-EC/DMC and **b,** 1M LiTFSI-FEC/FDMA electrolyte.

Supplementary Table 1. Highest occupied molecular orbital (HOMO)-lowest unoccupied molecular orbital (LUMO) energy values from density functional theory (DFT) simulations.

Salt/Solvent	HOMO (eV)	LUMO (eV)	Energy gap (eV)
**$LiPF_6$**	-11.000	-1.693	9.308
**LiFSI**	-9.157	-1.940	7.217
**LiTFSI**	-8.921	-1.709	7.212
**$LiNO_3$**	-7.708	-1.602	6.106
**LiBOB**	-7.739	-3.322	4.417
**EC**	-8.464	-0.602	7.862
**FEC**	-8.971	-0.641	8.330
**DMC**	-8.216	-0.235	7.981
**EMC**	-8.141	-0.219	7.922
**FDMA**	-7.549	-0.826	6.723
**DMA**	-6.729	-0.459	6.270
**DMF**	-6.888	-0.471	6.417
