# Supplementary material for: Interface chemistry of an amide electrolyte for highly reversible lithium metal batteries
Source: Nat Commun. 2020 Aug 21;11:4188. doi: 10.1038/s41467-020-17976-x (PMC7442789; doi:10.1038/s41467-020-17976-x)
Supplement: Supplementary file 2 — Description of Additional Supplementary Files [file 41467_2020_17976_MOESM2_ESM.pdf]

Description of Additional Supplementary Files

Supplementary Movie 1

Description: The morphology evolution for Li-metal anode cycling in 1M $LiPF_6$-EC/DMC with in-situ optical microscopy. Uneven Li deposition and porous morphology can be observed in platting process, and a large amount of residual Li remains on the surface after Li stripping.

Supplementary Movie 2

Description: The morphology evolution for Li-metal anode cycling in 1M LiTFSI-FEC/FDMA with in-situ optical microscopy. Homogeneous nucleation and denser Li deposition can be observed, and almost all the Li deposits disappear after Li stripping.
